# Wearable Heart Rate Monitoring Device Communicating in 5G ISM Band for IoHT

**DOI:** 10.3390/bioengineering10010113

**Published:** 2023-01-12

**Authors:** Ilaria Marasco, Giovanni Niro, Suleyman Mahircan Demir, Lorenzo Marzano, Luca Fachechi, Francesco Rizzi, Danilo Demarchi, Paolo Motto Ros, Antonella D’Orazio, Marco Grande, Massimo De Vittorio

**Affiliations:** 1 Department of Electrical and Information Engineering, Politecnico di Bari, 70125 Bari, Italy; 2 Center for Biomolecular Nanotechnologies, Istituto Italiano di Tecnologia, 73010 Arnesano, Italy; 3 Department of Electronics and Telecommunications, Politecnico di Torino, 10129 Turin, Italy; 4 Department of Engineering and Innovation, Università del Salento, 73100 Lecce, Italy

**Keywords:** wearable devices, sub-6GHz 5G, ISM band, piezoelectric sensor, heart rate monitoring, IoHT, integrated clinical environment

## Abstract

Advances in wearable device technology pave the way for wireless health monitoring for medical and non-medical applications. In this work, we present a wearable heart rate monitoring platform communicating in the sub-6GHz 5G ISM band. The proposed device is composed of an Aluminium Nitride (AlN) piezoelectric sensor, a patch antenna, and a custom printed circuit board (PCB) for data acquisition and transmission. The experimental results show that the presented system can acquire heart rate together with diastolic and systolic duration, which are related to heart relaxation and contraction, respectively, from the posterior tibial artery. The overall system dimension is 20 mm by 40 mm, and the total weight is 20 g, making this device suitable for daily utilization. Furthermore, the system allows the simultaneous monitoring of multiple subjects, or a single patient from multiple body locations by using only one reader. The promising results demonstrate that the proposed system is applicable to the Internet of Healthcare Things (IoHT), and particularly Integrated Clinical Environment (ICE) applications.

## 1. Introduction

With the maturity of wearable technologies, the devices able to measure heartbeats are becoming more widespread in daily use [1]. Several scientific studies demonstrate the correlation between heart rate (HR) and some clinical diseases [2,3], increasingly drawing the attention of both industry and end users to these devices. The gold standard as regards HR measurements is electrocardiography (ECG) [4]. However, ECG sensors, such as chest-strap monitors, are less attractive than other solutions because of their size and wearability [5]. A technology combining low-power consumption, high wearability, and the ability to convert the heartbeat derived from a mechanical deformation to an electrical signal can be extremely attractive for HR measurements. In this sense, piezoelectric sensors come to aid [6,7]. Natta et al. [8] presented a flexible AlN piezoelectric sensor. The sensor is passive and presents a very high sensitivity despite a compact footprint. Therefore, it can be exploited for measuring heartbeat through vein deformations in a non-invasive way. Moreover, integration of this technology with RF systems can pave the way for a new class of wireless low-power consuming and highly sensitive devices in the field of IoHT and sub-6GHz 5G-spectrum applications [9,10], where several sensor nodes are wirelessly connected for medical reasons [11,12,13,14]. The standard *de facto* as regards wireless sensors is represented by Bluetooth (BT) and ZigBee technologies. These present several advantages, such as long-range data transmission (theoretically up to 100 m in the outdoor scenario using Bluetooth), high data rate, and plenty of supported devices [15,16,17,18]. However, the ISM dedicated channels are overpopulated by other transmission standards, such as Wi-Fi and this can introduce interference problems [19]. Another issue is related to the attenuation due to water absorption which makes it difficult to establish radio links longer than 10 or 20 m in wet environments [20]. Moreover, synchronization of multiple devices is challenging [21]. Furthermore, the use of these standards is not suitable for real-time communications as the information is subdivided into packets [22]. This can be critical for ICE applications in which the availability of the data has to be guaranteed ceaselessly with the highest possible fidelity.

In this work, we propose a wearable sensing platform for HR monitoring working in the sub-6GHz 5G ISM band. This frequency range represents an optimal trade-off between all the bands available worldwide in terms of antenna’s footprints, bandwidth, and communication range. Indeed, the frequencies below 2.4 GHz have been discarded because the lower is the transmitting frequency the larger is the footprint of the antennas, and the narrower is the reserved band. The band around 2.4 GHz suffers of the problem of overpopulation since there are plenty of communication standards working in that range. Finally, the frequencies higher than 6 GHz are not suitable for long-range communications (mm-waves) due to the higher attenuations and coherence problems.

Furthermore, the proposed device has been used to transfer data from multiple sensors proving the possibility of monitoring several subjects continuously. Moreover, when multiple transmitting devices are enabled, the system does not require synchronization, in contrast with Bluetooth, thanks to real-time data streaming. This also provides the opportunity of using the same device for monitoring a single patient from multiple body locations. In addition, the proposed device is characterized by very high connection robustness. Moreover, it presents a small footprint, low-power consumption despite long-range data transmission, lightweight, and high-profile wearability. All these aspects can be crucial advantages for communications in medical applications from a future perspective [23].

## 2. System Design and Characterization

**Figure 1:**
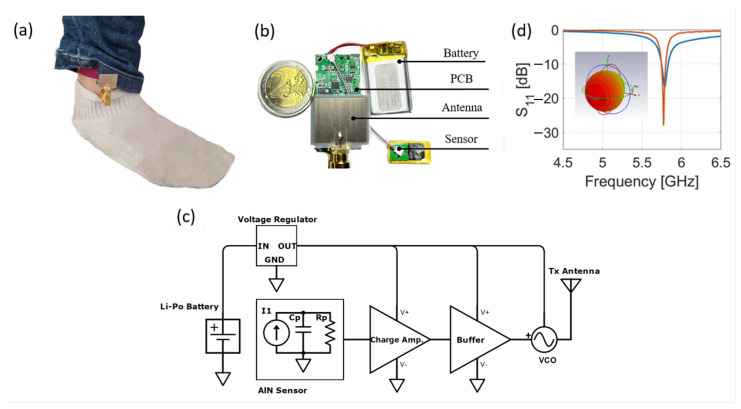
System overview: (**a**) subject wearing the proposed device, (**b**) breakdown of the system components, (**c**) simplified schematic of the PCB, and (**d**) simulated return loss and radiation pattern of the patch antenna.

The wearable system shown in Figure 1a,b is composed of a biocompatible AlN sensor, a custom PCB, and a patch antenna. The PCB, which has an overall dimension of 20 mm by 40 mm, consists of a charge amplifier, a buffer stage, and a voltage-controlled oscillator (VCO), depicted in Figure 1c. Artery pulses cause deformations of the piezoelectric sensor which are translated into an electrical signal because of the piezoelectric effect [24]. The sensor is connected to the PCB with a 50 mm-long U-FL to U-FL cable. On the board, the charge output is converted into a voltage with a gain of 1 mV/pC by the charge amplifier, which also introduces a high-pass filter with a cut-off frequency of 0.67 Hz. The converted voltage is applied to a VCO and modulates an RF signal whose carrier frequency ($f_{c}$) is tuned in the range of interest (between 5.725 GHz and 5.875 GHz) applying a bias voltage through a buffer stage. The RF modulated signal is transmitted by a compact patch antenna (14 mm × 16 mm) realized on a Polyethylene Naphtalate (PEN) flexible substrate. The antenna is connected to the board through SMA connectors. Figure 1d reports the simulated and measured return loss and the simulated radiation pattern at 5.8 GHz. The platform is powered by a standard 3.7 V 250 mAh Li-Po battery (5 mm × 20 mm × 30 mm).

**Figure 2:**
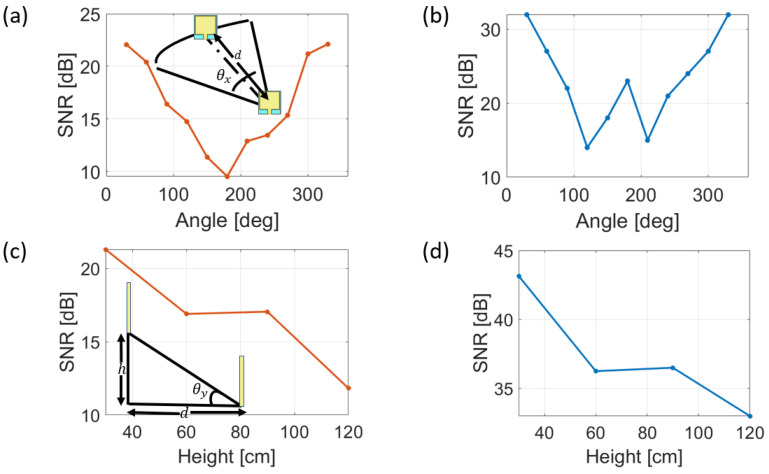
Power signal to noise ratio (SNR) at different conditions: variation of angles between antennas (**a**) outdoor scenario and (**b**) indoor scenario; different heights between antennas (**c**) outdoor scenario and (**d**) indoor scenario.

The sensing platform has been characterized by analysing the signal-to-noise ratio (SNR) defined in (1) [25]:

(1) $$SNR=10\log\frac{P_{signal}}{P_{noise}}$$

where $P_{signal}$ is the power of the received signal and $P_{noise}$ is the noise level. The measurement has been performed for various distances and angles between the transmitting and the receiving antennas in indoor and outdoor scenarios. The results are presented in Figure 2.

Figure 2a,b show the trend of the measured SNR obtained by placing the antennas at a 1-m distance, and changing the planar angle between them from 0 to 360° detailed for outdoors and indoors scenarios, respectively. For the outdoor scenario, the data are in agreement with the simulated radiation pattern reported in Figure 1d as there is a minimum at 180° in Figure 2a. For the indoor scenario, Figure 2b, it is still possible to observe a symmetric behaviour of the SNR with respect to 180°: it decreases from 0° to 100° and increases locally at 180°. This effect is related to the presence of walls that reflect the signal during its propagation.

Further analysis concerning the variation of the vertical angle between antennas has been carried out for both environments. In more detail, keeping the antennas at 1 m of distance, the height has been swept from 0.3 to 1.2 m corresponding to a variation of the vertical angle between 16.7° and 50.2°. In these cases, the results shown in Figure 2c,d are very similar, as the SNR decreases with the increase in the relative height between the antennas.

Finally, the maximum communication distance has been evaluated. In [25], the minimum SNR for a detectable signal is reported as 10 dB; for the proposed system, this threshold is reached at 55 m for the indoor scenario. Further analysis has been performed in terms of weight and power consumption. It results that the device is lightweight (about 20 g) and has the power consumption of 100 mW (3.7 V and 27 mA).

## 3. Results and Discussions

**Figure 3:**
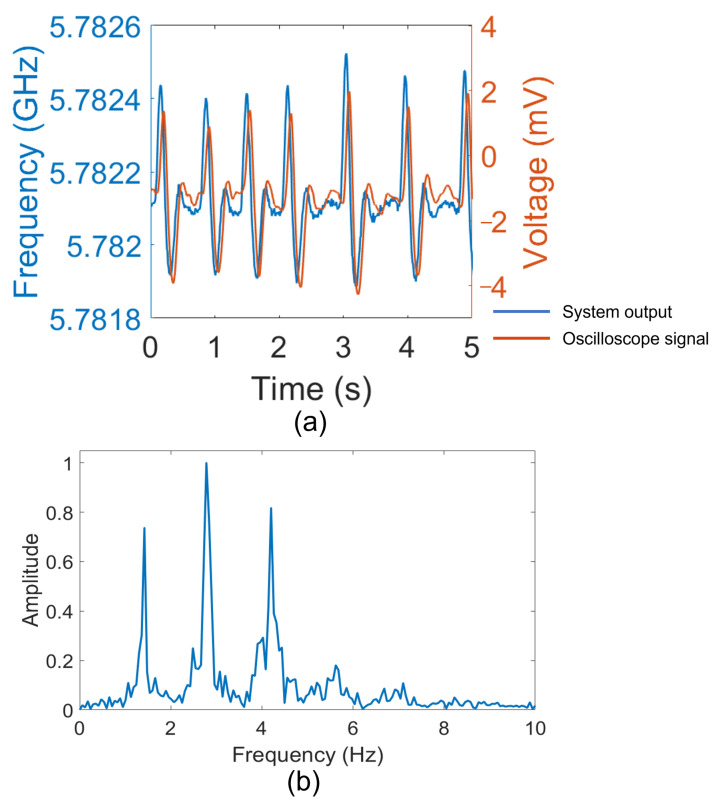
Measurement results: (**a**) output of the system (blue curve), compared with respect to a reference signal taken from the oscilloscope (red curve); (**b**) the Fast Fourier Transform of the demodulated signal.

The signal transmitted from the system (see blue curve of Figure 3a) represents the response of the piezoelectric sensor due to the pulsation of the posterior tibial artery and is presented in a graph of “frequency vs time” where the amplitude is given by the bandwidth (BW) of the signal. The signal has been acquired with the subjects in rest position and has been compared with the response of the piezoelectric sensor (see red curve of Figure 3a) when connected to a benchtop commercial charge amplifier (the charge amplifier Kistler LabAmp Type 5165A) having the same settings (gain and offset) of the board and measured with an oscilloscope (MDO4104-3-Tektronix). It is worth stressing that only a qualitative comparison of the shape can be performed since the amplitude of the signal from the oscilloscope is expressed in millivolts (mV) while the output of our system is in Gigahertz (GHz), due to the frequency modulation. It is possible to note that the two signals appear clear and with a very low level of noise in both cases. It results that no differences appear in the peak shapes obtained with the oscilloscope or our wireless system. The shape clearly identifies the heartbeats and is very similar to the signal reported in [26]. Then, using the Fast Fourier Transform (FFT) considering the total time of the signal of 15 s acquired with a sampling frequency of 100 Hz, important heart-related parameters such as the HR based on the number of beats per minute (bpm), and the mean duration of the diastolic (heart relaxation) and systolic (heart contraction) phases have been extrapolated. Figure 3b shows the results of the analysis. The signal in the frequency domain highlights three peaks in defined intervals considering the volunteer subject is at rest position. In particular, the peak observed between 0.8 Hz and 1.5 Hz refers to the HR, which could be extracted in terms of bpm by multiplying the value by 60. The peak associated with the diastole, which lasts about 0.4 s, is found between 2 and 3 Hz; the duration of this phase is not affected by the duration of the heart cycle. On the other hand, the systole has a duration of about 0.3 s which highly depends on the duration of the cardiac cycle. At rest, it is possible to find the peak between 3 Hz and 5 Hz, but this frequency could increase in case of higher heart rates that correspond to a shorter duration of the cycle.

**Figure 4:**
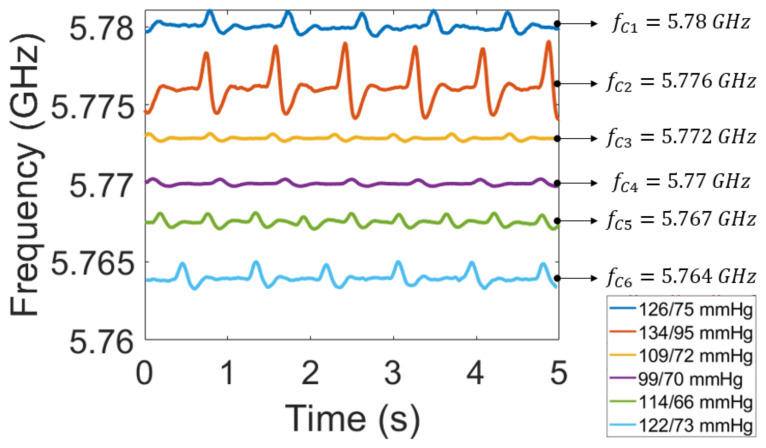
Simultaneous heartbeat signals from six volunteer subjects.

The analysis performed by the proposed system on the single volunteer has been extended to the monitoring of six healthy subjects, males and females, with ages between 25 and 29 years old, by using the same reader. In order to simultaneously acquire multiple signals, the carrier frequency of each transmitter has been set by just modifying the VCO bias voltages. In this regard, Figure 4 shows the heartbeat signals of the different subjects recorded at the same time. It is possible to observe that among the six signals there are some differences and some similarities, depending on the considered signals. In particular, the amplitude concerning subject 2 (blue) is higher with respect to the others, while there are more peaks for subject 5 that can be associated with a higher heart rate. Instead, the difference in amplitude can be attributed to the blood pressure; it is related to the pushing force of the blood against the artery that leads to higher deformations and consequently to a higher charge generated by the piezoelectric sensor. In this regard, a medically validated device, OMRON M3 Comfort (HEM-7154-E) Blood Pressure Monitor [27], has been used for the measurement of the blood pressure of the subjects. The values measured for subjects 1 and 6 are almost the same, 126/75 mmHg and 122/73 mmHg, respectively, and it can be noticed that they are comparable in amplitude (1.61 MHz for subject 1 and 1.47 MHz for subject 6). On the other hand the pressure of subject 2 is significantly higher, 134/95 mmHg, leading to a significantly wider signal of 3.8 MHz. In addition, measures of subjects 3 and 4, characterized by lower values of blood pressures, are reported. The result follows the expectation since an amplitude lower than 0.5 MHz is observed.

Table 1 details the comparison between our proposal and the state of the art in wireless heart monitoring systems. As it can be noted, our approach represents a good compromise between transmission distances and power consumption. Moreover, our system avoids the problem of channel congestion, characterizing the 2.4 GHz-band, exploiting the new 5G ISM band. The use of this band represents an optimal trade-off in the quest for a wide bandwidth (150 MHz), a small electrical wavelength (50 mm), which reduces the footprint of the antenna. It is worth stressing that at the ISM radio band in which this system works, the bandwidth is 150 MHz while the maximum bandwidth of the signals is about 4 MHz. According to the presented results, a maximum signal bandwidth of 5 MHz, and a frequency span of 1 MHz between each signal can be considered, leading, theoretically, to a simultaneous monitoring of 25 subjects.

## 4. Conclusions

In this work, we present a wearable wireless sensing device working in the sub-6GHz 5G ISM band for the continuous monitoring of heart rate and duration of diastolic and systolic phases of a cardiac cycle from the posterior tibial artery. The characterization of the system demonstrates that the device can work effectively even if the transmitting and receiving antennas are located at different heights and angles. Further analysis has been performed for one to six volunteers to demonstrate the functionality of the system. Considering the available ISM band and the maximum frequency span of the signals, a total number of 25 subjects that can be simultaneously monitored with a single reader has been mathematically estimated. In the future steps, we will define the maximum number of subjects monitorable and we will improve our system by adapting it to acquire the heart rate from different body locations of a single subject to contribute IoHT, ICE, and Wireless Body Area Network applications.

## Figures and Tables

**Table 1:** Results comparison with the other reported works.

	Sensor Type	Sensor Location	Comm. Type	Central Freq. (GHz)	ISM Bandwidth (MHz)	Max. Indoor Distance (m)	Tx Power (dBm)
[18]	Thermo-sensation	Wrist	Digital	2.4 (BLE)	100	<100	0
[15]	Piezoelectric	Neck	Digital	0.868 (ZigBee)	5	<100	20
[28]	Piezoelectric (PVDF)	Wrist	Digital	2.4 (BT)	100	<100 *	20 *
[17]	MEMS Pressure	Wrist	Digital	2.4 (BT)	100	<100 *	20 *
[16]	Piezoelectric	In-ear	Analog	2.4	100	NA	0
[29]	RFID Tag	Chest	Analog	5.8 (5G ISM)	150	NA	35
This Work	Piezoelectric (AlN)	Ankle	Analog	5.8 (5G ISM)	150	55	2

* The values are estimated based on the utilized technologies reported by the papers.

## Data Availability

Not applicable.

## References

[1] Windmiller J.R., Wang J. (2013). Wearable Electrochemical Sensors and Biosensors: A Review. Electroanalysis.

[2] Heimrich K.G., Lehmann T., Schlattmann P., Prell T. (2021). Heart rate variability analyses in Parkinson’s disease: A systematic review and meta-analysis. Brain Sci..

[3] Kant N., Peters G.M., Voorthuis B.J., Groothuis-Oudshoorn C.G., Koning M.V., Witteman B.P., Rinia-Feenstra M., Doggen C.J. (2022). Continuous vital sign monitoring using a wearable patch sensor in obese patients: A validation study in a clinical setting. J. Clin. Monit. Comput..

[4] Serhani M.A., T. El Kassabi H., Ismail H., Nujum Navaz A. (2020). ECG monitoring systems: Review, architecture, processes, and key challenges. Sensors.

[5] Kwon S.H., Dong L. (2022). Flexible Sensors and Machine Learning for Heart Monitoring. Nano Energy.

[6] Sezer N., Koç M. (2021). A comprehensive review on the state-of-the-art of piezoelectric energy harvesting. Nano Energy.

[7] Demir S.M., Al-Turjman F., Muhtaroğlu A. (2018). Energy scavenging methods for WBAN applications: A review. IEEE Sens. J..

[8] Natta L., Guido F., Algieri L., Mastronardi V.M., Rizzi F., Scarpa E., Qualtieri A., Todaro M.T., Sallustio V., De Vittorio M. (2021). Conformable AlN Piezoelectric Sensors as a Non-invasive Approach for Swallowing Disorder Assessment. ACS Sens..

[9] Marasco I., Niro G., Rizzi F., Vittorio M., D’Orazio A., Grande M. Design of a PEN-Based Flexible PIFA Antenna Operating in the sub-6GHz Band for 5G Applications. Proceedings of the 2020 22nd International Conference on Transparent Optical Networks (ICTON).

[10] Marasco I., Niro G., Rizzi F., Vittorio M., D’Orazio A., Grande M. (2022). A compact evolved antenna for 5G communications. Sci. Rep..

[11] Kim S., Pakzad S., Culler D., Demmel J., Fenves G., Glaser S., Turon M. Health monitoring of civil infrastructures using wireless sensor networks. Proceedings of the 6th International Conference on Information Processing in Sensor Networks.

[12] Ketu S., Mishra P.K. (2021). Internet of Healthcare Things: A contemporary survey. J. Netw. Comput. Appl..

[13] Gubbi J., Buyya R., Marusic S., Palaniswami M. (2013). Internet of Things (IoT): A vision, architectural elements, and future directions. Future Gener. Comput. Syst..

[14] Niro G., Marasco I., Lamanna L., Rizzi F., D’Orazio A., de Vittorio M., Grande M. Fabrication of a Flexible Film Bulk Acoustic Resonator for Wireless Sensor Networks. Proceedings of the 2022 Microwave Mediterranean Symposium (MMS).

[15] Al Ahmad M., Ahmed S. Heart-rate and pressure-rate determination using piezoelectric sensor from the neck. Proceedings of the 2017 4th IEEE International Conference on Engineering Technologies and Applied Sciences (ICETAS).

[16] Park J.H., Jang D.G., Park J.W., Youm S.K. (2015). Wearable sensing of in-ear pressure for heart rate monitoring with a piezoelectric sensor. Sensors.

[17] Kang X., Zhang J., Shao Z., Wang G., Geng X., Zhang Y., Zhang H. (2022). A Wearable and Real-Time Pulse Wave Monitoring System Based on a Flexible Compound Sensor. Biosensors.

[18] Fu Y., Zhao S., Zhu R. (2018). A wearable multifunctional pulse monitor using thermosensation-based flexible sensors. IEEE Trans. Biomed. Eng..

[19] Union I.T. (2012). Radio Regulations Articles. ITU Radio Regul..

[20] Christoe M.J., Yuan J., Michael A., Kalantar-Zadeh K. (2021). Bluetooth signal attenuation analysis in human body tissue analogues. IEEE Access.

[21] Coviello G., Florio A., Avitabile G., Talarico C., Wang-Roveda J.M. (2022). Distributed Full Synchronized System for Global Health Monitoring Based on FLSA. IEEE Trans. Biomed. Circuits Syst..

[22] Jara A.J., Fern’ndez D., López P., Zamora M.A., Ubeda B., Skarmeta A.G. Evaluation of bluetooth low energy capabilities for continuous data transmission from a wearable electrocardiogram. Proceedings of the 2012 Sixth International Conference on Innovative Mobile and Internet Services in Ubiquitous Computing.

[23] Bayramzadeh S., Aghaei P. (2021). Technology integration in complex healthcare environments: A systematic literature review. Appl. Ergon..

[24] Martin R.M. (1972). Piezoelectricity. Phys. Rev. B.

[25] Mazda F. (2014). Telecommunications Engineer’s Reference Book.

[26] Li P., Karmakar C., Liu C., Liu C. Analysing effect of heart rate and age on radial artery pressure derived systolic and diastolic durations in healthy adults. Proceedings of the 2015 Computing in Cardiology Conference (CinC).

[27] Omron Healthcare (2010). M3 Comfort Upper Arm Blood Pressure Monitor.

[28] Xin Y., Qi X., Qian C., Tian H., Ling Z., Jiang Z. (2014). A Wearable Respiration and Pulse Monitoring System Based on PVDF Piezoelectric Film. Integr. Ferroelectr..

[29] Mishra A., Li C. (2019). A low power 5.8-GHz ISM-band intermodulation radar system for target motion discrimination. IEEE Sens. J..

